# Single and Repeated Administration of Methylphenidate Modulates Synaptic Plasticity in Opposite Directions via Insertion of AMPA Receptors in Rat Hippocampal Neurons

**DOI:** 10.3389/fphar.2018.01485

**Published:** 2018-12-19

**Authors:** Claudia Carvallo, Darwin Contreras, Gonzalo Ugarte, Ricardo Delgado, Floria Pancetti, Carlos Rozas, Ricardo Piña, Luis Constandil, Marc L. Zeise, Bernardo Morales

**Affiliations:** ^1^Laboratory of Neuroscience, Faculty of Chemistry and Biology, Universidad de Santiago de Chile, Santiago, Chile; ^2^Department of Biology, Faculty of Sciences, Universidad de Chile, Santiago, Chile; ^3^Laboratory of Environmental Neurotoxicology, Faculty of Medicine, Universidad Católica del Norte, Coquimbo, Chile; ^4^School of Psychology, Faculty of Humanities, Universidad de Santiago de Chile, Santiago, Chile

**Keywords:** methylphenidate, AMPA receptor, synaptic plasticity, hippocampus, LTP

## Abstract

Methylphenidate (MPH) is widely used in the treatment of Attention Deficit Hyperactivity Disorder. Several lines of evidence support that MPH can modulate learning and memory processes in different ways including improvement and impairment of test performances. A relevant factor in the efficacy of treatment is whether administration is performed once or several times. In this study we demonstrate opposite effects of MPH on performance of preadolescent rats in the Morris Water Maze test. Animals treated with a single dose (1 mg/kg) performed significantly better compared to controls, while in animals treated with repetitive administration at the same concentration performance was reduced. We found that hippocampal LTP in slices from rats treated with a single dose was increased, while LTP from rats treated with repetitive injections of MPH was lower than in controls. Using Western blot of CA1 areas from potentiated slices of rats treated with a single dose we found a significant increase of phosphorylation at Ser845 of GluA1 subunits, associated to an increased insertion of GluA1-containing AMPARs in the plasma membrane. These receptors were functional, because AMPA-dependent EPSCs recorded on CA1 were enhanced, associated to a significant increase in short-term plasticity. In contrast, CA1 samples from rats injected with MPH during six consecutive days, showed a significant decrease in the phosphorylation at Ser845 of GluA1 subunits associated to a lower insertion of GluA1-containing AMPARs. Accordingly, a reduction of the AMPA-mediated EPSCs and short-term plasticity was also observed. Taken together, our results demonstrate that single and repeated doses with MPH can induce opposite effects at behavioral, cellular, and molecular levels. The mechanisms demonstrated here in preadolescent rats are relevant to understand the effects of this psychostimulant in the treatment of ADHD.

## Introduction

Methylphenidate (MPH), an amphetamine derivative, is widely prescribed for the treatment of Attention Deficit Hyperactivity Disorder (ADHD) and increasingly used as a drug of abuse by students (Sahakian and Morein-Zamir, 2007; Weyandt et al., 2016). Its pharmacodynamics is similar to those of amphetamine and cocaine (Volkow et al., 1995; Solanto, 2000). MPH is an effective treatment, but its potential long-term effect from an early age on in brain development is unclear, and long-term consequences may be possible (Marco et al., 2011). The consequences of stimulant exposure are increased synaptic levels of dopamine and norepinephrine in several brain regions including the hippocampus and the prefrontal cortex (PFC) (Kuczenski and Segal, 2001; Berridge and Devilbiss, 2011). Psychostimulant-induced hippocampal alteration has been implicated in the therapeutic potential as well as the long-term risk of this type of medication (Britton and Bethancourt, 2009).

The effects of MPH on cognitive functions in humans as well as in animal models are highly contentious. Several studies show that repeated administration of MPH can impair cognitive functioning: Heyser et al. (2004) found recognition memory impairment in young animals administering MPH (5 mg/kg; i.p.) twice daily for 7 days. Scherer et al. (2010) demonstrate that chronic exposure to a therapeutic dose of MPH (2.0 mg/kg) in juvenile rats significantly impairs spatial learning/memory, as assessed in the Morris water maze (MWM). Another study found that rats exposed to 3 or 5 mg/kg administered for 11 or 21 days exhibited no significant preference for exploration of the novel object, suggesting that chronically administered MPH induces impairment of recognition memory in rats (LeBlanc-Duchin and Taukulis, 2007). On the other hand, repeated administration of MPH also can improve cognitive performance. Thus, low orally administered doses of MPH (0.5 mg/Kg) improve spatial working memory performance in rats in a T maze, while higher doses induce perseverative errors (Devilbiss and Berridge, 2008; Spencer et al., 2015). Thus, low doses of MPH seem to enhance, while high doses to impair cognitive functions.

Whether cognitive functions are boosted, impaired or remain untouched also depends on age, as well as the cognitive domain considered (Linssen et al., 2014). Further, an important parameter, sometimes underestimated in our opinion, is whether the substance is administered once or in a chronic manner. Curiously, in humans, even though in therapy the typical case is repetitive administration, investigations in healthy human volunteers are mainly performed as single-dose studies, while animal behavioral studies typically are performed repetitively, administering MPH a short while (such as half an hour) before each test trial. Almost no studies exist comparing acute and chronic administration are (however, see Sadasivan et al., 2012).

An important question in this context is the duration of psychostimulant effects. It is well known that repeated administration of psychostimulants can cause changes in cognitive performance lasting for days up to life-long changes. Thus, chronic administration of cocaine or amphetamine in early development of rats has been found to cause functional changes in the nervous system that may translate into behavioral and cognitive deficits (Robinson and Kolb, 2004). This includes impaired performance on learning measures, such as recognition and spatial memory (Bisagno et al., 2003). Lagace et al. (2006) demonstrated that 2.0 mg/kg MPH administered twice daily in preadolescent rats for 15 days interferes with neurogenesis, possibly leading to enduring changes.

Further, studies with adolescent rats have found behavioral evidence suggesting potentially MPH-induced changes in brain function that persist long after administration of the drug has been completely excreted from the system (Eckermann et al., 2001; Bolaños et al., 2003; Mague et al., 2005). Recently, we found that MPH (1 mg/kg) induced an increase in LTP in the prefrontal cortex of rats that was even more pronounced 18 days later than immediately following daily administration for 15 days (Burgos et al., 2015).

In the present study we investigated the effects of a single administration of MPH (1 mg/kg) compared to a repetitive one at the same dose, demonstrating changes in visuo-spatial learning and plasticity that lasted more than 24 h. An administration of 1 mg/Kg MPH i.p. in rats results in 40 ng/ml in plasma, concentration equivalent to the one reached with clinical doses used in the treatment of the ADHD in adolescent humans (Swanson and Volkow, 2003; Swanson et al., 2011). Our studies in hippocampal slices superfused with MPH have shown a dose-dependent LTP increase with an EC_50_ of 73.44 ± 6.32 nM equivalents to 17.13 ng/ml, a value of same order as the 40 ng/ml measured in plasma (Rozas et al., 2015).

Investigating the cellular and molecular mechanisms of the effects of MPH on plasticity, the involvement of catecholamine receptors has been demonstrated. Both, α2 adrenoceptors and dopaminergic D1 receptors, contribute to the enhancing effects of MPH on working memory in rodents (Arnsten and Dudley, 2005). In the prefrontal cortex (Berridge and Devilbiss, 2011) as well as in the hippocampus *in vivo* (Jenson et al., 2015) and in the slice (Rozas et al., 2015), the involvement of the two catecholamine receptor types in learning and LTP changes have been shown. We have proposed a polysynaptic model of the two receptor types acting in series. D1 receptor action raises intracellular cAMP levels at postsynaptic sites of glutamate synapses with consistent PKA activation. Finally, AMPARs regulation mediated by PKA promotes an increase of the LTP by promoting the trafficking and insertion of functional AMPARs into the plasma membrane (Rozas et al., 2015).

Here we present evidence that a certain dose (1 mg/kg) can have opposite effects on visuo-spatial learning and the cellular and molecular mechanisms underlying it, depending on its administration as single dose or repetitively. Single administration of MPH improved the learning performance, enhancing LTP by insertion of AMPA-GluA1 receptor subunits, while repeated administration impaired behavioral performance, decreasing the LTP due to a decreased number of AMPA-GluA1 receptor subunits in the plasma membrane.

## Experimental Procedures

### Animals

The care and procedures described below were approved by the Committee for bioethics of the Universidad de Santiago de Chile. Sprague-Dawley rats were bred and maintained under stable conditions of temperature and humidity, with food and water *ad libitum* under a 12 h light–dark cycle.

### Administration of Methylphenidate

In the group receiving administration once, 21 rats were injected with a dose of 1 mg/kg MPH (racemic hydrochloride) and 21 with saline (NaCl 0.9%) *i.p.*, administered 30 min before the first session. 30 min before each one of the following 5 sessions only saline was injected to the experimental as well as to the control group. Animals treated with a single dose of MPH and controls were sacrificed the day after the first and last session and hippocampal slices were prepared (see “Hippocampal Slices”).

In the repeated dose group MPH (1 mg/kg) or saline were given 30 min before each session for 6 days (21 individuals either group). The day after the last session hippocampal slices were prepared as described in Section “Hippocampal Slices.”

### Morris Water Maze

**FIGURE 1:**
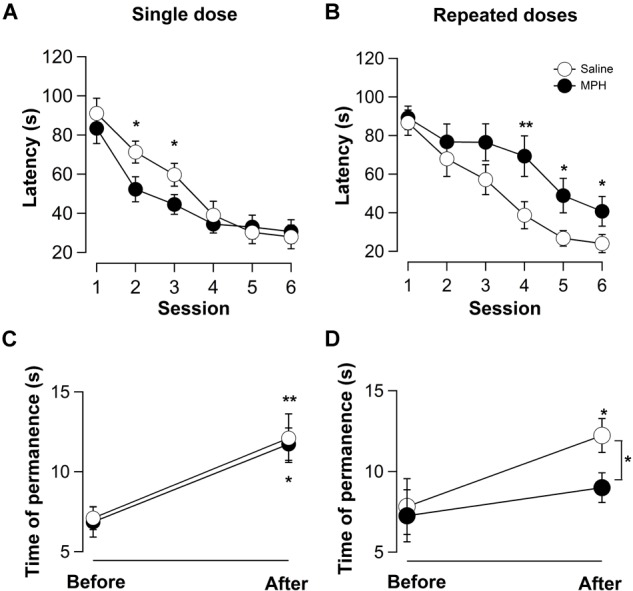
A single dose of methylphenidate (MPH) improves, while repeated doses impair the visuo-spatial learning in the Morris water maze (MWM). Average latencies for the first session compared with the last session were significantly different for all groups, suggesting the learning of the assigned task (*n* = 21, ^∗^*p* < 0.05). **(A)** Single dose of MPH facilitates visuo-spatial learning. For the 2nd and 3rd session latencies were significantly shorter in rats treated with MPH compared to controls, {two-way ANOVA [*F*_(5,219)_ = 25.53; ^∗^*p* < 0.05]; Bonferroni *post hoc* test (*n* = 21; ^∗^*p* < 0.05)}. **(B)** Time of permanence in the target quadrant significantly increased for either group indicating successful training (saline-treated: before 7.1 ± 0.7 s, after 12.1 ± 1.5 s, *n* = 21, ^∗^*p* < 0.05; MPH-treated: before 6.9 ± 0.9 s, after 11.7 ± 1.0 s, *n* = 21; ^∗∗^*p* < 0.01) with no significant difference between controls and treated rats (controls: 12.0 ± 1.5 s; MPH: 11.7 ± 1.0 s; *n* = 21; *p* > 0.05). **(C)** Repeated doses of MPH slowed visuo-spatial learning. In the 4th, 5th, and 6th sessions rats treated with MPH were slower than controls [two-way ANOVA: for drug treatment; *F*_(1,221)_ = 15.64, *p* < 0.001 and session; *F*_(5,221)_ = 17.97, *p* < 0.001]. **(D)** Repetitive administration of MPH impaired memory retrieval. Different from control animals (from 7.8 ± 1.7 s to 12.2 ± 1.5 s; *n* = 21; ^∗^*p* < 0.05), no significant difference between pre- and post-training was found in MPH-treated rats. The difference between controls and treated rats was significant (from 12.2 ± 1.0 s to 9.0 ± 0.9 s; *n* = 21; ^∗^*p* < 0.05). Saline-treated rats (open circles); MPH-treated rats (solid circles).

Behavioral training and testing were conducted in a circular pool (diameter 180 cm, depth 60 cm) painted black and half-way filled with water rendered opaque with white latex paint and maintained at a temperature of 24°C. Four geometrical figures in black and white (approximately 20 cm wide) attached to the upper rim of the pool and situated at an angle of 90° to each other, provided the optical cues, thus forming four quadrants. A white curtain surrounded the pool to obscure external cues. Animal movements were monitored with a video camera mounted above the pool. Behavioral data were recorded and analyzed using ANY-maze video tracking software (Stoelting Co., IL, United States). Two experimental trials were run each day 1 trial immediately following the other. The sessions in Figure 1 represent the average of the two trials per day.

Previous to the training the rats were allowed to swim for 30 s in the pool. During the six experimental sessions (2 trials each) an invisible platform (12 cm in diameter) was present, located 2 cm below water level at a distance of 20 cm from the pool’s edge in the middle of a quadrant. Launching positions were changed arbitrarily from session to session. Subjects were allowed to swim until they had placed all four paws on the platform or until 120 s had elapsed. Animals that did not get to the platform in 120 s were placed on it for further 30 s. Finally, 24 h after one more session without injecting was performed with the platform removed, measuring the time of permanence in the quadrant where the platform had been located during training and compared with the corresponding time before training (observation time: 30 s).

### Elevated Plus-Maze

Anxiety-related behavior was measured using the elevated plus-maze test. The maze consisted of two opposite open arms (50 cm × 10 cm), and two opposite arms at a right angle to the open arms with 40 cm high walls. The maze was elevated 50 cm above the floor. Each rat was placed for 5 min in the apparatus for acclimatization prior to starting the tests. Immediately after the pretest, the rats were placed in the center of the maze facing one of the open arms. The maze was cleaned after each trial with 70% ethanol.

Anxiety-related behavior was quantified comparing the total time spent in the open and closed arms, with the time spent in the closed arms. We also checked for the level of locomotor activity by measuring the average speed (cm/s).

Testing was performed at day 1 using the hidden platform immediately after the first MWM session and at day 6 after the last session.

### Hippocampal Slices

Rats were sacrificed by decapitation under isoflurane anesthesia. Hippocampi were dissected out and cut in transversal slices of 400 μm with a vibratome (Leica, Nussloch, Germany) in cold dissection buffer containing (in mM): 212.7 sucrose, 5 KCl, 1.25 NaHPO_4_, 3 MgSO_4_, 1 CaCl_2_, 26 NaHCO3, and 10 glucose (pH 7.4). The slices were transferred to a storage chamber kept at room temperature in artificial cerebrospinal fluid (ACSF) containing (in mM): 124 NaCl, 5 KCl, 1.25 NaH_2_PO_4_, 1 MgCl_2_, 2 CaCl_2_, 26 NaHCO_3_, and 10 glucose (pH 7.4, in 95% O_2_/5% CO_2_), and kept for at least 1 h before recording. In the recording chamber, hippocampal slices were superfused with ACSF at a rate of 1 mL/minute at 30°C.

### Field Excitatory Postsynaptic Potentials (fEPSPs)

Field excitatory postsynaptic potentials (fEPSPs) were recorded as previously described (Rozas et al., 2015, 2012). Briefly, the fEPSPs were evoked applying electrical stimulation from Schaeffer collateral commissural fibers and recorded in the stratum radiatum of the CA1 region. Test pulses were applied every 15 s. A baseline was established with test pulses adjusted to evoke 50% of the maximal response. After recording a stable baseline for at least 20 min, LTP was induced with theta burst stimulation (TBS, consisting of 5 trains of 10 bursts at 5 Hz each, 1 burst, 4 pulses at 100 Hz). In all experiments the fEPSP recordings were continued for 60 min after initiating TBS. The synaptic responses were quantified as the initial slope of evoked fEPSPs and plotted as percentage change referred to the average slope measured during baseline recording.

To analyze pre- and postsynaptic components of synaptic responses, we used the following paired-pulse stimulation protocol: 2 pulses with an interstimulus interval of 50 ms were applied every 15 s, 20 min before and 50 min after TBS. The results are presented as the ratio between the initial slopes of fEPSP evoked by the second and the first stimulus (paired pulse ratio; PPR). This measure reflects the quantal release of neurotransmitter from presynaptic components (Schulz et al., 1994). If the drug modifies the paired pulses facilitation (PPF) curve, it can be concluded that it acts at a presynaptic level.

### Whole-Cell Recording

For whole-cell voltage clamp recording, CA1 neurons were visually identified with an infrared differential interference contrast microscope (Zeiss, Oberkochen, Germany). Patch pipettes (2–4 MΩ) were filled with internal solution containing (in mM): 130 cesium gluconate, 2 ATP-Mg, 8 KCl, 10 EGTA, 10 HEPES, and 1 QX-314, pH 7.4 (CsOH, 275–285 mOsm). The junction potential (typically <5 mV) was compensated. Only cells with membrane potentials more negative than -65 mV, access resistance <20 MΩ (8–18 MΩ, compensated at 80%), and input resistance >100 MΩ (130–410 MΩ) were studied. Cells with input or access resistance changing more than 15% during the experiment were discarded. All recordings were performed at 28–30°C. The electrically evoked AMPAR-mediated excitatory postsynaptic currents (EPSCs) were recorded at holding potentials of -65 mV. Bathing solution was ACSF supplemented with 10 μM picrotoxin in order to block GABA_A_-dependent currents.

### Western Blot Analysis

CA1 areas of hippocampal slices used in electrophysiological studies were dissected and homogenized in 1 mL of lysis buffer containing (in mM): 150 NaCl, 50 NaF, 10 NaPPi, 1 NaVO_3_, 5 EDTA, 10 EGTA, 20 NaPO_4_, pH 7.4, containing protease inhibitors (Halt^TM^ Protease Inhibitor Cocktail, Thermo Scientific, Rockford, IL, United States).

The homogenates were centrifuged at 13,560 rpm for 10 min at 4°C and the supernatants were collected and stored at -20°C. Protein concentrations were determined using the BCA^TM^ protein assay kit (Thermo Scientific, Rockford, IL, United States). Samples of 50 μg were run on 12% polyacrylamide gels, and transferred to nitrocellulose membranes for Western blot analysis. Primary antibodies were purchased from Millipore (Temecula, CA, United States) and used in the following dilutions: rabbit anti-GluR-1 (1:500), rabbit anti-phospho GluR-1-Ser845 (1:1000), rabbit anti-phospho GluR-1-Ser831 (1:1000). After incubation with HRP-conjugated secondary antibodies (1:10,000; Thermo Scientific, Rockford, IL, United States), reactive proteins were visualized using chemiluminescent substrates (Thermo Scientific, Rockford, IL, United States). GluA1 phosphorylation at Ser845 was quantified using Image J software normalizing band intensity to the total GluA1-associated bands for each gel individually.

### BS3-Crosslinking Assay

CA1 areas from hippocampal slices used in electrophysiological recordings were then removed and incubated in ACSF solution containing bis(sulfosuccinimidyl) suberate (BS3) (1 mg/mL; SIGMA, IL, United States) with gentle agitation for 30 min on ice (Boudreau and Wolf, 2005; Boudreau et al., 2012). The crosslinking reaction was quenched with 100 mM glycine. BS3-crosslinked and non-crosslinked paired samples were fractionated and 40 μg of protein were charged and run in SDS-PAGE using 4 and 15% gradient gels (Bio-Rad) and blotted as described below.

Surface and intracellular pools of GluA-1 subunit in the samples were evaluated quantifying the band intensities associated with high molecular weight crosslinked complexes (tetrameric form) and non-crosslinked proteins (monomeric form) in paired BS3-treated and untreated samples, using Image J software. The fraction of surface receptors for each sample was calculated as the ratio between band intensity associated with surface-associated crosslinked receptors and the total amount of receptors (intensity of monomeric GluA-1 associated bands in non-crosslinked samples) for each gel individually.

### Drugs

Methylphenidate hydrochloride was kindly donated by Laboratorio Andrómaco (Santiago, Chile). FSK and SKF38393 were purchased from Sigma (St. Louis, MO, United States).

### Data Analysis

Results of the MWM test were analyzed using Student’s *t*-test and the general linear model ANOVA (fixed factor) for repeated (intragroup) and independent (intergroup) testing as appropriate, followed by the Bonferroni comparison test. Electrophysiological data are presented as mean ± SEM and normalized relative to the baseline (average slope of fEPSPs measured before the TBS protocol). The “*n*” values given in parentheses correspond to the number of animals and recorded slices, respectively. LTP was measured during the final 20 min of the recording, and presented as the averaged percentage of baseline. Statistical significance for mean differences between two experimental and control groups was assessed using Student’s *t*-test and Mann–Whitney *U*-test.

The statistical significance of each Western blot-assay crosslinking gel was evaluated by means of mean differences between experimental and control groups using Mann–Whitney *U*-test.

## Results

### Effect of Single and Repeated Treatment of MPH on Visuo-Spatial Learning and Memory

In order to determine the effect of single and repeated administration of MPH on visuo-spatial learning and memory retrieval, the MWM test was performed. Figures 1A,B show that the latencies to reach the escape platform decreased significantly from the 1st to the 6th session, and with a similar time course during the 6 sessions in the all the experimental groups, showing that each group of rats learned the assigned task efficiently (*n* = 21, ^∗^*p* < 0.05). A significant decrease in the time to reach the platform from 71.3 ± 5.6 s to 52.3 ± 6.4 s, and from 59.7 ± 5.8 s to 44.7 ± 5.0 s, was found in the 2nd and 3rd sessions between the control rats and the rats treated once with MPH, respectively (*n* = 21; ^∗^*p* < 0.05; Student’s *t*-test; Figure 1A). These results were confirmed with a two-way ANOVA [*F*_(5,219)_ = 25.53; ^∗^*p* < 0.05, Bonferroni *post hoc* test, ^∗^*p* < 0.05]. This suggests that a single dose of treatment of MPH improves visuo-spatial learning.

In contrast, a significant increase in the latencies from 38.7 ± 7.0 s to 69.3 ± 10.5 s during the 4th session (*n* = 21; ^∗∗^*p* < 0.01; Student’s *t*-test), from 26.7 ± 4.0 s to 48.9 ± 9.0 s in the 5th session (*n* = 21, ^∗^*p* < 0.05) and from 24.0 ± 4.7 to 40.7 ± 7.7 s in the 6th session (*n* = 21, ^∗^*p* < 0.05), were observed in repeatedly treated rats with MPH compared to the controls (Figure 1C). These results were confirmed by two-way ANOVA analysis, showing significant differences by drug treatment [*F*_(1,221)_ = 15.64, *p* < 0.001, Bonferroni *post hoc* test, ^∗^*p* < 0.05] and session [*F*_(5,221)_ = 17.97, *p* < 0.001, Bonferroni *post hoc* test, ^∗^*p* < 0.05], suggesting that the repeated treatment with MPH impaired visuo-spatial learning.

To further evaluate the effect of MPH on memory retrieval, we measured the time spent in each quadrant of the MWM before and after training with the platform removed. In rats treated with a single dose of MPH a significant increase was found in the average time of permanence in the target quadrant: saline (from 7.8 ± 1.7 s to 12.2 ± 1.5 s; *n* = 21; ^∗^*p* < 0.05) and MPH (from 6.9 ± 0.9 s to 11.7 ± 1.0 s, *n* = 21; ^∗∗^*p* < 0.01), suggesting that all rats could retrieve memory about the place where the platform had been situated (Figure 1B). However, in these rats no significant difference was found in the time spent in the quadrant between saline-treated rats and MPH-treated rats: from 12.0 ± 1.5 s to 11.7 ± 1.0 s (*n* = 21; *p* > 0.05). This suggests that a single dose of MPH does not change retrieval of the visuo-spatial memory 6 days after administration (Figure 1B). However, in the group of repeatedly treated rats, only the controls displayed a significant increase in the time spent in the target quadrant during training trials (from 7.8 ± 1.7 s to 12.2 ± 1.5 s; *n* = 21; ^∗^*p* < 0.05; Figure 1D), whereas the animals who had received MPH repeatedly did not. The difference between controls and treated rats was significant from 12.2 ± 1.0 s to 9.0 ± 0.9 s (*n* = 21; ^∗^*p* < 0.05), suggesting that repetitive treatment with MPH impairs the memory retrieval in a visuo-spatial task (Figure 1D).

Taken together, a single dose of MPH improves visuo-spatial learning, without change in memory retrieval, while the repeated treatment of MPH impairs visuo-spatial learning and memory retrieval. These findings cannot be explained by differences in swimming speed nor anxiety, since no significant difference was found between the control and treated groups with a single dose of MPH [swim: *F*_(1,60)_ = 0.499, *p* = 0,482; anxiety: days 1 and 6 *F*_(1,36)_ = 0.258, *p* = 0,614, Bonferroni *post hoc* test, *p* > 0.05] nor with repeated administration [swim: *F*_(1,60)_ = 0.132, *p* = 0.718; anxiety: days 1 and 6 *F*_(1,36)_ = 0.506, *p* = 0.50, Bonferroni *post hoc* test, *p* > 0.05], suggesting that neither single nor repeated treatment with MPH used in this study affected locomotor activity or anxiety (Supplementary Figure S1). Moreover, these results cannot be associated to the stress level induced by manipulation since no significant difference was found in corticosterone levels between repeatedly treated and control rats with or without testing in the MWM (Supplementary Figure S2).

### Effect of Single and Repeated Treatment With Methylphenidate on the TBS-Induced Hippocampal LTP

**FIGURE 2:**
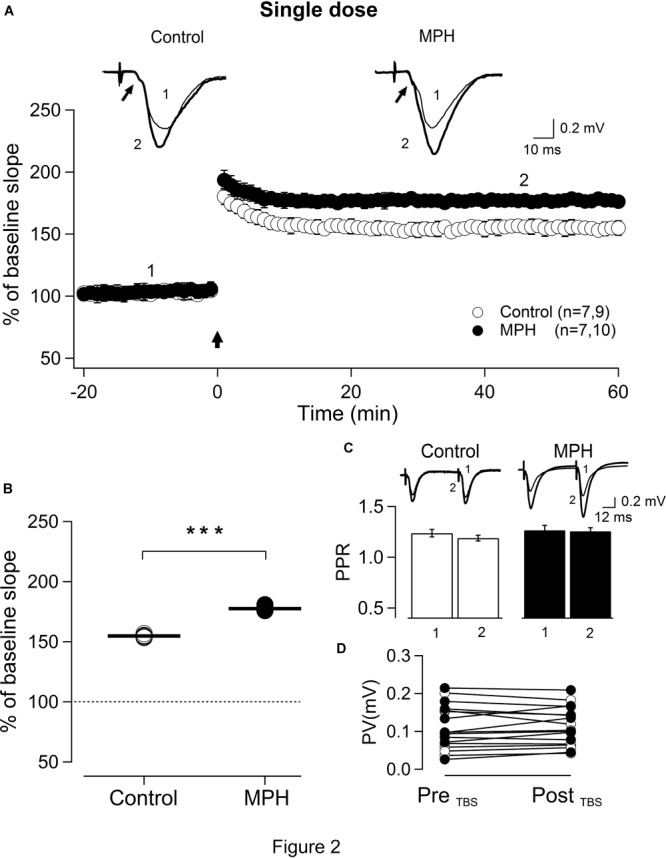
Methylphenidate administered as a single dose increases LTP in the hippocampal CA1 area. **(A)** Time course of the TBS-induced LTP in slices prepared from saline-treated rats (open circles) vs. rats treated with a single dose of MPH (solid circles). Insets: representative recordings averaging 10 traces at times marked 1 and 2. Small arrow: presynaptic volley. Parentheses: number of animals and recorded slices, respectively. Vertical arrow indicates TBS. **(B)** Control LTPs (154.7 ± 0.4%; *n* = 7, 9; baseline: 100%), vs. LTPs from treated rats (177.5 ± 0.6%; *n* = 7,10; ^∗∗∗^*p* < 0.001). **(C)** Paired-pulse ratios were not significantly different for any condition including post-TBS between saline-treated (2; white columns) and MPH-treated rats (2; black columns; *n* = 10, 10; *p* > 0.05) indicating mainly postsynaptic mechanisms. Inset: synaptic responses at times marked 1 and 2 in A in slices prepared from saline-treated (white columns) vs.- MPH-treated rats (black columns). **(D)** Relationships of the presynaptic fiber volley magnitude (PV) before (Pre) and after (Post) TBS were not significantly different. Control (open circles): 0.102 ± 0.021 mV (Pre_TBS_), 0.097 ± 0.017 mV (Post_TBS_) (*n* = 9, *p* > 0.05). MPH (solid circles): 0.118 ± 0.020 mV (Pre_TBS_), 0.127 ± 0.017 mV (Post_TBS_) (*n* = 10, *p* > 0.05). Each symbol represents one experiment.

In order to determine the relationship between the behavioral effects and LTP induction, fEPSPs were recorded in hippocampal slices derived from control rats and treated ones. Figure 2A shows LTP recordings from slices prepared after the second session of rats treated with a single dose of saline and 1 mg/kg of MPH, respectively. Consistent with our behavioral studies, MPH increased slightly, but significantly, the magnitude of the LTP induced by TBS from 154.7 ± 0.4% in the controls (*n* = 7,9) to 177.5 ± 0.6% in the treated rats (*n* = 7,10; ^∗∗∗^*p* < 0.001; Figure 2B). No difference between LTPs in controls vs. treated rats was found in slices obtained 24 h after the last session corresponding to 6 days after the injection (Supplementary Figure S3).

**FIGURE 3:**
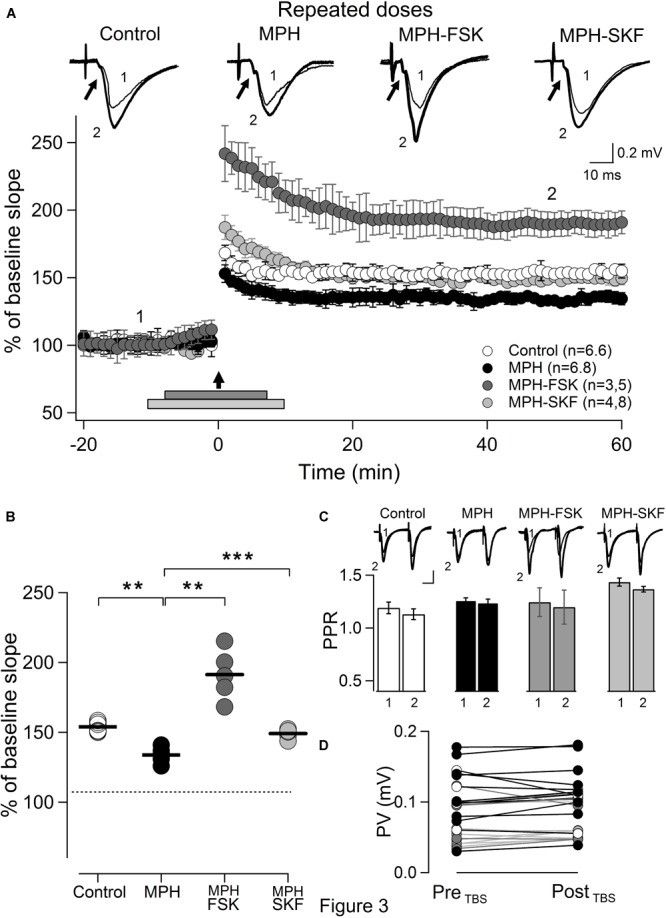
Repeated administration of MPH decreases LTP in the hippocampal CA1 area. Symbols and marks as in Figure 2; **(A)** TBS-induced LTPs in slices from rats treated repetitively with: Saline (open circles) or MPH (solid circles); slices from MPH-treated rats superfused from 7 min before until 7 min after TBS (bar) with 50 μM Forskolin (MPH-FSK; dark gray circles); or from 10 min before until 10 min after TBS with 5 μM SKF38393 (MPH-SKF; light gray circles). Parentheses: number of animals, recorded slices. Arrow indicates TBS. Inset: recordings averaging 10 traces, under the four conditions at times marked 1 and 2. Small arrow: presynaptic fiber volley (PV). **(B)** Quantification of LTPs. Decrease of 20% between saline (control) and MPH-treated rats (MPH) from 154 ± 1.5% (*n* = 6,6) to 133.8 ± 2.1% (*n* = 6,8; ^∗∗^*p* < 0,05), reverted with 50 μM FSK, an adenylyl cyclase activator, from 133.8 ± 2.1% to 191.3 ± 8.0% (MPH-FSK; *n* = 3,5; ^∗∗∗^*p* < 0.001); and with 5 μM SKF38393, a D1/D5 receptor agonist, from 133.8 ± 2.1 to 149.2 ± 0.9 (MPH-SKF; *n* = 4,8; ^∗∗∗^*p* < 0.001). **(C)** Paired-pulse ratios (PPRs). Color code for columns as in **(A)**. Inset: synaptic responses evoked by two stimulations applied with interstimulus intervals of 50 ms at times marked 1 and 2 in the experiments in **(A)**. No significant difference in PPRs for any condition. Control: 1.19 ± 0.06 (1) vs. 1.13 ± 0.05 (2) (*n* = 10,10; *p* > 0.05). MPH: 1.26 ± 0.03 (1) vs. 1.24 ± 0.04 (2) (*n* = 10,10; *p* > 0.05). MPH-FKS: 1.25 ± 0.14 (1) vs. 1.21 ± 0.16 (2) (*n* = 3,5; *p* > 0.05). MPH-SKF: 1.44 ± 0.04 (1) vs. 1.37 ± 0.03 (2) (*n* = 4,8; *p* > 0.05). **(D)** As Figure 2D. No significant difference was found. Control (open circles): 0.103 ± 0.001 mV (Pre_TBS_), 0.101 ± 0.008 mV (Post_TBS_) (*n* = 7, *p* > 0.05). MPH (solid circles): 0.113 ± 0.018 mV (Pre_TBS_), 0.120 ± 0.017 mV (Post_TBS_) (*n* = 8, *p* > 0.05). MPH-FSK (dark gray circles): 0.080 ± 0.017 mV (Pre_TBS_), 0.081 ± 0.014 mV (Post_TBS_) (*n* = 5, *p* > 0.05). MPH-SKF (light gray circles): 0.054 ± 0.006 mV (Pre_TBS_), 0.059 ± 0.005 (Post_TBS_).

**FIGURE 4:**
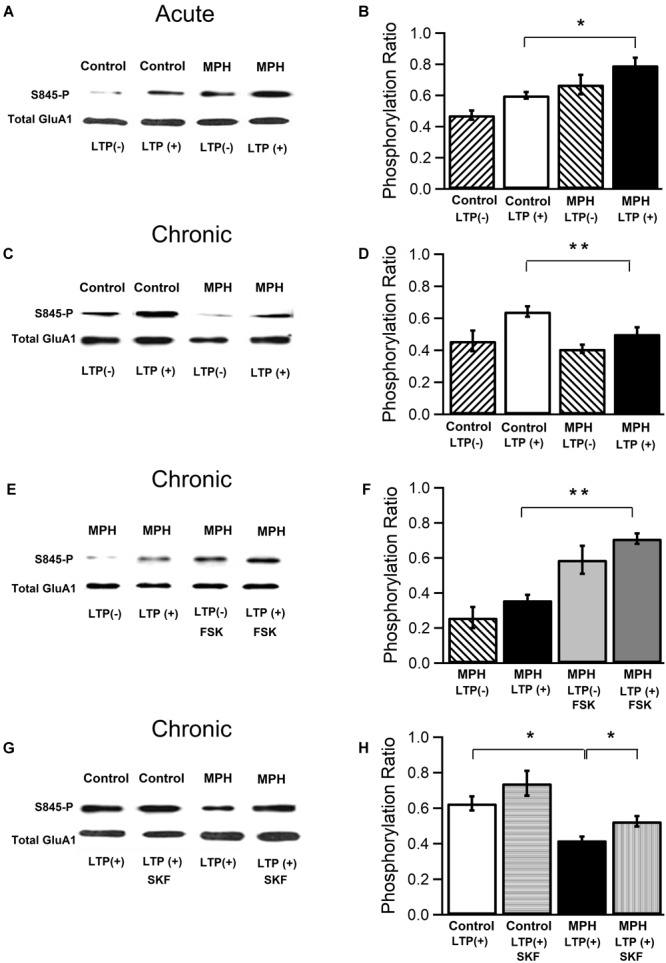
Effect of single and repeated administration of methylphenidate on the phosphorylation of the AMPA receptor GluA1 subunit. From CA1 areas of hippocampal slices used in LTP experiments (Figures 2, 3) total protein extracts were prepared and analyzed using Western blot. **(A,B)** LTP increase induced by a single dose of MPH is associated with an increase of the phosphorylation of serine 845 at the AMPAR GluA1 subunit. **(A)** Immunoblot using antibody against the phosphorylated Ser845 (PKA site) of GluA1: Control LTP (–) = slices from naïve rats, Control LTP (+) = slices from saline-treated rats, MPH LTP(–), MPH LTP(+) = slice from MPH-treated rats, and antibody recognizing the C-terminal end of GluA1 (total GluA1). **(B)** Quantification of Ser845 phosphorylation ratio 60 min after TBS. Control LTP (–): 0.47 ± 0.03; Control LTP (+): 0.60 ± 0.02; MPH LTP (–): 0.67 ± 0.06; MPH LTP (+): 0.79 ± 0.05 (*n* = 5,5; ^∗^*p* < 0.05). **(C,D)** Reduction of LTP induced by repeated administration of MPH is associated with a decrease in the serine 845 phosphorylation of the AMPAR GluA1 subunit. **(C)** As in **(A)**, but after repeated doses. **(D)** As in **(B)**, but after repeated MPH administration. Control LTP (–): 0.46 ± 0.06; Control LTP (+): 0.64 ± 0.03; MPH LTP (–): 0.41 ± 0.03; MPH LTP (+): 0.50 ± 0.04 (*n* = 5,5; ^∗∗^*p* < 0.01). **(E,F)** Forskolin (FSK; 50 μM) reverted the decrease in the serine 845 phosphorylation at the AMPAR GluA1 subunit os in slices from rats repeatedly treated with MPH. **(E)** As in **(C)**, but all measurements from repeatedly MPH-treated animals without and with forskolin present **(F)** As in **(D)**, but from rats repeatedly treated with MPH without and with 50 μM forskolin. MPH LTP (–): 0.26 ± 0.06; MPH LTP (+): 0.36 ± 0.03; MPH LTP(–)-FSK: 0.59 ± 0.08; MPH LTP(+)-FSK: 0.71 ± 0.03 (*n* = 4,4; ^∗∗^*p* < 0.01). **(G,H)** SKF38393 (SKF; 5 μM) superfused during 20 min reverted the decrease in serine 845 phosphorylation from rats treated repeatedly with MPH. **(G)** As in **(E)**, but superfusing slices with SKF. **(H)** As in **(F)** but superfused with 5 μM SKF. Control LTP (+): 0.63 ± 0.04; Control LTP (+)-SKF: 0.74 ± 0.04; MPH LTP(+): 0.42 ± 0.02; MPH LTP(+)-SKF: 0.53 ± 0.03 (*n* = 3,4; ^∗^*p* < 0.05).

In contrast, recordings from slices prepared 24 h after the last session from rats that showed impairment in the visuo-spatial learning induced by repeated treatment with MPH, presented a significant decrease in the magnitude of TBS-dependent LTP from 154 ± 1.5% (saline; *n* = 6,6) to 133.8 ± 2.1% (MPH; *n* = 6,8) (^∗∗^*p* < 0.05; Figures 3A,B). Since MPH acts through D1/D5 receptors activating the cAMP-PKA pathway (Rozas et al., 2015), we performed experiments demonstrating its functionality by superfusing slices of rats repeatedly MPH-treated, with 50 μM forskolin, an adenylyl cyclase activator, applied 7 min before until 7 min after TBS. The LTPs in these slices increased from 133.8 ± 2.1% (*n* = 6,8) to 191.3 ± 8.0% (*n* = 3,5; ^∗∗^*p* < 0.05; Figures 3A,B). In line with this idea, we superfused slices during 20 min with SKF38393, a D1/D5 receptor agonist, at a concentration of 5 μM. SKF38393 failed to induce changes in basal electrical activity or TBS-dependent LTP (Supplementary Figure S4). 5 μM of SKF reverted the decrease of LTP observed in rats repeatedly treated with MPH from 133.8 ± 2.1 to 149.2 ± 0.9 (*n* = 4,8; ^∗∗∗^*p* < 0.001; Figures 4A,B). No significant difference was observed in the LTP magnitude in slices from saline-treated rats (from 154 ± 1.5% to 150.2 ± 3.8%; *n* = 4,8; *p* > 0.05; Figures 4A,B). These results are consistent with the idea that the LTP reduction observed with repeatedly administered MPH is due to an effect on D1/D5 receptors.

Neither the facilitation nor the decrease of MPH-dependent LTP were attributable to changes in the recruitment of presynaptic fibers, since no significant change was observed in the magnitude of the presynaptic fiber volley under any condition (arrows in traces of Figures 2A,D, 3A,D, respectively).

### LTPs Induced After Single and Repeated Treatment of MPH Involve Modification of Post-synaptic Components

To find out whether the changes induced by single and repeated treatment of MPH are pre- or postsynaptic in CA3–CA1 synapses, we assessed the contribution of presynaptic terminals employing paired-pulse protocols on slices obtained 24 h after the 1th session and 6th session, respectively. Figure 2C shows that slices prepared from rats 24 h after a single dose of MPH display no significant difference in the paired pulse ratio (PPR) of 50 ms before (1 filled column) and after (2 filled columns) TBS (1.27 ± 0.05 before TBS vs. 1.26 ± 0.04 after TBS; *n* = 7,10; *p* > 0.05). As expected, we did not find significant differences in the PPR of slices from saline-treated rats either (1.24 ± 0.04 before LTP vs. 1.19 ± 0.03 after LTP; *n* = 7,9; *p* > 0.05). Similar results were obtained in slices from rats treated repeatedly with MPH. No significant difference was found in the paired pulse ratio of 50 ms before (1 open column) and after (2 open column) TBS between treated rats (1.26 ± 0.03 before LTP vs. 1.24 ± 0.04 after MPH-facilitated LTP; *n* = 7,10; *p* > 0.05) and control rats (1.19 ± 0.06 before LTP vs. 1.13 ± 0.05 after TBS-dependent LTP; *n* = 7,9; *p* > 0.05; Figure 3C).

We also tested whether the effects of forskolin and SKF38393, are pre- or postsynaptic. Although an increase in the presynaptic volley amplitude and a depression in the PPF were seen during the superfusion with forskolin, this effect was only transient since 30 min after drug washout these were not different from baseline values [1.25 ± 0.14 before LTP (1; dark gray column) vs. 1.21 ± 0.16 during LTP (2; dark gray column); *n* = 3,5; *p* > 0.05; Figure 3C], confirming that the late effect of forskolin is postsynaptic in hippocampal CA1 (Otmakhov et al., 2004). On the other hand, we also failed to observe a significant difference in the PPR in slices prepared from repeatedly treated rats superfused with SKF38393 [1.44 ± 0.04 before LTP (1; light gray column) vs. 1.38 ± 0.03 during LTP (2; light gray column); *n* = 4,8; *p* > 0.05; Figure 3C].

These results suggest that neither LTP increase induced by single dose MPH nor its reduction by repeatedly applied MPH nor the effect of forskolin or SKF38393 does involve modifications of presynaptic components in the CA3–CA1 synapse, but are rather due to changes at the postsynaptic level.

### Effect of Single and Repeated Treatment of Methylphenidate on the Phosphorylation of the AMPA Receptor GluA1 Subunit

We assessed the role of the serine 845 residue (PKA site) of the GluA1 subunit of AMPAR in single and repeated treatment with MPH. Our experimental approach was to collect CA1 areas from hippocampal slices used in electrophysiological experiments and to analyze the phosphorylation state at the Ser845 residue of the GluA1 subunit by the Western blot technique. CA1 areas from slices that exhibited an enhanced TBS-dependent LTP after single dose administration with MPH (177.5 ± 0.6%) displayed a significant increase of GluA1 phosphorylation at the Ser845 residue (PKA site) compared to those with TBS-dependent LTP without MPH treatment (154.7 ± 0.4%) at 60 min after TBS (from 0.60 ± 0.02 to 0.79 ± 0.05; *n* = 5,5; df = 3; ^∗^*p* < 0.05; *F* = 13; Figures 4A,B). No difference between controls vs. treated rats was found 6 days after the injection (Supplementary Figure S3). In contrast, and consistent with our electrophysiological results, repeated treatment with MPH induced a significant decreased in the phosphorylation state of the Ser 845 residue in GluA1 subunits from 0.64 ± 0.03 to 0.50 ± 0.04 for control LTPs and MPH-dependent LTPs, respectively (*n* = 4,4; df = 3; ^∗∗^*p* < 0.05; *F* = 8,7; Figures 4C,D). In slices from rats repeatedly treated with MPH a significant increase of LTP with 50 μM forskolin was observed, together with a reversion in the phosphorylation level of Ser 845 from 0.36 ± 0.03 to 0.71 ± 0.03 (*n* = 4,4; df = 7; ^∗∗^*p* < 0.01; Figures 4E,F). Further, a recovery in the phosphorylation levels from 0.42 ± 0.02 to 0.53 ± 0.03 was observed with SKF38393, in those slices from MPH-repeated treated rats that showed a return to a normal state of LTP (*n* = 3,4; df = 7; ^∗^*p* < 0.05; Figures 4G,H).

### Effect of Single and Repeated Treatment With Methylphenidate on the Insertion of GluA1 Subunit Into the Plasmatic Membrane

**FIGURE 5:**
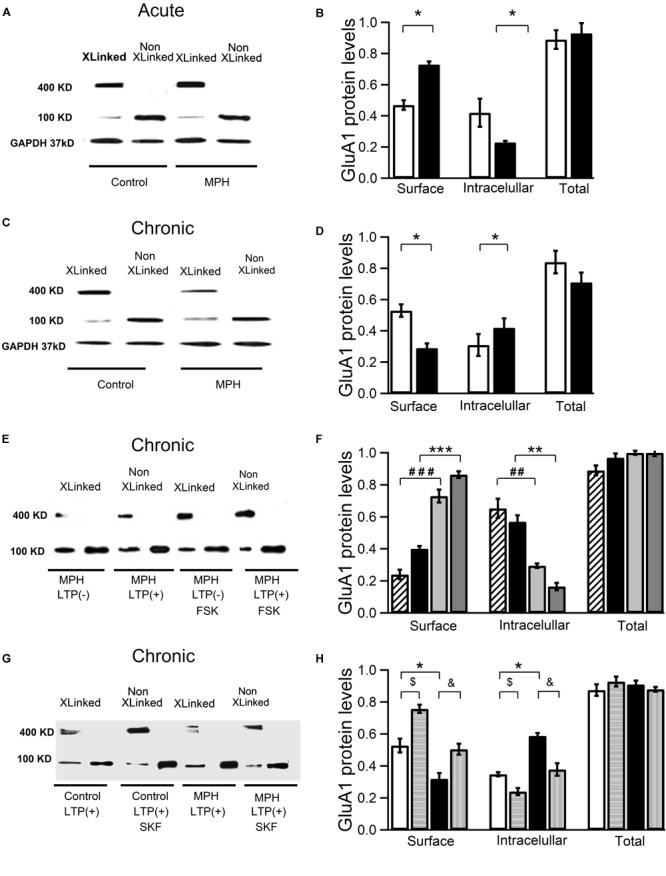
Methylphenidate given as single dose increases LTP whereas repeated MPH administration decreases LTP *via* changes in the number of AMPA receptors in the membrane. CA1 areas excised from hippocampal slices used in the electrophysiological experiments were taken to perform crosslinking assays and inmunoblots using an antibody against GluA1 subunits. (A,B) LTP increase induced by a single dose of MPH is associated with an increase in the insertions of GluA1 subunits in the cell surface. **(A)** Immunoblot using an antibody against GluA1 subunits in samples prepared from slices of saline-treated rats (control) and MPH-treated rats (MPH) that presented LTP. Levels of GluA1 total proteins were calculated as the sum of surface (S) and intracellular (I) protein levels. Samples treated (Xlinked) and untreated (non-Xlinked) with BS3. 400 kD bands indicate surface-expressed GluA1 subunits, 100 kD bands intracellular GluA1 subunits. **(B)** After single dose administration, the fraction of surface-associated GluA1 subunits increases from 0.47 ± 0.03 in controls to 0.73 ± 0.02 in slices of MPH-treated rats (*n* = 4,8; ^∗^*p* < 0.05); correspondingly the fraction of the intracelullar pool decreases from 0.42 ± 0.09 to 0.23 ± 0.01 (*n* = 4,8; ^∗^*p* < 0.05). **(C,D)** In slices from repeatedly treated rats AMPAR GluA1 insertion in the cell surface was reduced. **(C)** As in **(A)**, but after repeated MPH administration. **(D)** Surface-associated GluA1 subunits are reduced from 0.53 ± 0.04 in controls to 0.29 ± 0,02 (*n* = 4,8; ^∗^*p* < 0,01), with a significant increase in the intracelullar pool fraction from 0.31 ± 0.07 to 0.42 ± 0.06, respectively (*n* = 4,8; ^∗^*p* < 0.05). **(E,F)** Superfusion with FSK; 50 μM) for 14 min reverted this drop in the number of surface-associated GluA1 subunits. **(E)** As in **(C)**, but from slices without or with foskolin superfusion (K). **(F)** As in D but comparing measurements without and with FSK. The fraction of surface-associated GluA1 subunits increased from 0.40 ± 0.02 in slices prepared from MPH-treated rats with LTP(+) (black columns) to 0.86 ± 0,02 in slices prepared from MPH-treated rats with LTP (+) additionally superfused with 50 μM FSK (dark gray columns; *n* = 3,5; ^∗∗∗^*p* < 0.001) with a significant decrease in the fraction of the intracelullar pool from 0.57 ± 0.04 to 0.17 ± 0.01, respectively (*n* = 4,4; ^∗∗^*p* < 0.01). There was also a priming effect in the insertion of AMPAR induced by FSK: Surface-associated GluA1 subunits significantly increased from 0.24 ± 0.03 in slices without TBS of MPH-treated rats (diagonal stripes columns) to 0.73 ± 0.04 in slices without TBS of MPH-treated rats but superfused with FSK (light gray columns) (*n* = 3,5; ^###^*p* < 0.01). This increase was consistent with the decrease in the fraction of the intracelullar pool: from 0.65 ± 0.06 to 0.30 ± 0.01 (*n* = 3,5; ##*p* < 0.01). **(G,H)** SKF38393 (5 μM; for 20 min) reverted the drop in the number of surface-associated GluA1 subunits **(G)** As in **(E)**, but superfusing SKF38393 SKF, **(H)** as in **(F)**, but without or with SKF38393 Surface-associated GluA1 subunits significantly decreased from 0.53 ± 0.04 in slices prepared from saline-treated rats and LTP(+) (white columns) to 0.320 ± 0,04 in slices prepared from MPH-treated rats with LTP (+) (dark columns) (*n* = 4,4; ^∗^*p* < 0.05); the fraction of the intracelullar GluA1 pool increased from 0.34 ± 0.01 to 0.58 ± 0.02, respectively (*n* = 4,4; ^∗^*p* < 0.05). This effect was reverted with SKF (vertical stripes column) (from 0.320 ± 0.04 to 0.505 ± 0.03; *n* = 4,4; ^&^*p* < 0.05) with a decrease in the intracelullar GluA1 pool (from 0.588 ± 0.01 to 0.378 ± 0.04; ^&^*p* < 0.05); the fraction of surface-associated GluA1 subunits increased (white columns, from 0.53 ± 0.043 to 0.76 ± 0.024; *n* = 4.4; ^$^*p* < 0.05) with SKF (horizontal stripes column); finally the fraction of the intracelullar GluA1 pool decreased with SKF (from 0.35 ± 0.019 to 0.24 ± 0.021; ^$^*p* < 0.05).

In order to determine whether single vs. repeated administrations of MPH induce changes in the number of AMPAR GluA1 subunits in the post-synaptic membrane of the CA1 area of the hippocampus, a crosslinking assay was performed to detect changes in receptor surface expression produced by a prior *in vivo* treatment. In line with our electrophysiological results, those slices that showed increased LTP (177.5 ± 0.6%) compared to the controls (154.7 ± 0.4%) also significantly increased the fraction of cell-surface-associated GluA1 subunits: from 0.47 ± 0.03 to 0.73 ± 0.02 (*n* = 4,8; df = 5; ^∗^*p* < 0,05; *F* = 49) as evidenced by the presence of high molecular weight crosslinked subunits (Figure 5A). As we expected, the pool of intracellular receptors declined from a ratio of 0.42 ± 0.09 in samples from slices with LTP(+) to 0.23 ± 0.01 in samples from slices with LTP + MPH (*n* = 4,8; ^∗^*p* < 0.05; Figures 5A,B). These results suggest that a single dose of MPH mobilizes GluA1 subunits from the intracellular pool to the surface 6 days after the single injection no significant difference between controls vs. treated rats was found anymore (Supplementary Figure S3).

In contrast, the slices from rats treated repeatedly with MPH with reduced LTP (137.4 ± 0.4%) compared to the controls (154.0 ± 0,1%) displayed a significant decrease in the fraction of cell-surface-associated GluA1 subunits from 0.53 ± 0.04 to 0.29 ± 0.03 (*n* = 4,4; df = 5; ^∗^*p* < 0.05; *F* = 46). On the other hand, the pool of intracellular receptors changed from 0.31 ± 0.07 in samples from control slices to 0.42 ± 0.06 in samples from slices of MPH-treated rats (*n* = 4,4; ^∗^*p* < 0.05; Figures 5C,D). The reduction of GluA1 subunits at the cell surface was also significantly reverted when the slices were superfused with 50 μM forskolin, from 0.40 ± 0.02 (black column) to 0.86 ± 0.02 (dark gray column) (*n* = 3,5; ^∗∗∗^*p* < 0.001; Figures 5E,F). Consistent with this, the pool of intracellular receptors changed from 0.57 ± 0.04 to 0.17 ± 0.01 (*n* = 4,4; ^∗∗^*p* < 0.01; Figures 5E,F). A recovery in the number of GluA1 subunits at the cell surface also was found with superfusion of SKF38393 from 0.32 ± 0.04 to 0.50 ± 0.03 consistent with the decrease in the fraction of the intracelullar pool (from 0.58 ± 0.01 to 0.37 ± 0.04; *n* = 4,4; ^∗^*p* < 0.05; Figures 5G,H).

Taken together, our results suggest that a single dose of MPH increases whereas repeated administration reduces the insertion of GluA1 subunits into the membrane surface.

### Single and Repeated Treatment With MPH Changes the AMPA-EPSC and Short-Term Plasticity in CA1 Hippocampal Neurons in Opposite Directions

**FIGURE 6:**
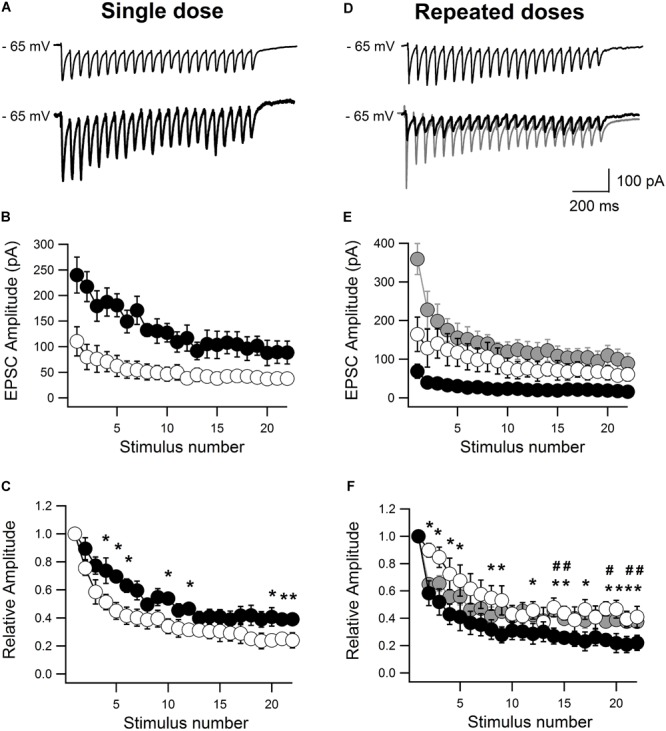
Single administration of MPH increases whereas repeated administration decreases the AMPA-mediated current and short-term plasticity. Using whole-cell recording AMPA-mediated EPSCs of pyramidal neurons from the CA1 area were evoked by 22 pulses at 20 Hz applied from Schaeffer collaterals at holding potentials of –65 mV. EPSCs were measured as current peak. **(A)** EPSCs averaged from five traces obtained in neurons of slices prepared from rats treated with saline only (thin trace) and treated with MPH once (thick trace). **(B)** Quantification of EPSCs. All EPSCs from treated rats (black circles) were significantly larger than those from controls (open circles; *n* = 5,5; ^∗∗^*p* < 0.01). **(C)** Short-term plasticity, measured as relative amplitude, i.e., as ratio of the *n*th to the first pulse, between recordings from treated (having received a single dose; black circles) compared to untreated rats (open circles) was strongly increased (*n* = 5,5; ^∗^*p* < 0.05). **(D)** As in **(A)**, but from rats treated with saline only (thin trace), treated repeatedly with MPH (thick trace) and treated repeatedly with MPH in the presence of 50 μM of FSK (gray trace; 7 min before and 7 min after TBS). **(E)** Quantification of EPSCs. All EPSCs from treated rats (black circles) were significantly smaller than those from controls (open circles; *n* = 4,5; ^∗^*p* < 0.05). FSK restored the size of EPSCs (gray circles; *n* = 3.5; ^∗^*p* < 0.05). **(F)** Short-term plasticity, measured as relative amplitude, ratio of the *n*th to the first pulse, between EPSCs of slices obtained from untreated (black circles) and treated rats (open circles) with repeated doses of MPH (*n* = 5,5; ^∗^*p* < 0.05). The decrease of short-term plasticity was partially reverted with 50 μM of FSK (gray circles: *n* = 3,5; ^#^*p* < 0.05).

Finally, we examined the functionality of the AMPARs that remain in the membrane after single and repeated treatments with MPH, studying the short-term plasticity in response to stimulus trains of 22 pulses at 20 Hz. AMPA-EPSCs were evoked by current stimulation on Schaeffer collaterals and recorded in CA1 pyramidal cells kept at -65 mV. The neurons of slices derived from single dose-treated rats showed an average amplitude of AMPA currents that was significantly higher than that of saline-treated rats for all the responses evoked by the stimulation protocol (*n* = 5,5; ^∗∗^*p* < 0.01; Figures 6A,B). In line with this, we found a significant increase in short-term plasticity, measured as the ratio of the currents evoked by *n*th vs. 1st pulse, in neurons from MPH-treated rats compared to controls (*n* = 5,5; ^∗^*p* < 0.05; Figure 6C). Conversely, in slices from repeatedly treated rats with MPH, AMPA-mediate EPSCs and short-term plasticity were significantly lower than in controls (*n* = 4,5; ^∗^*p* < 0.05; Figures 6D–F). This effect was reverted by superfusing those slices 7 min before until 7 min after TBS with 50 μM of forskolin (*n* = 3,5; ^#^*p* < 0.05; Figures 6D–F). These results suggest that the increase and decrease of short-term plasticity induced by single and repeated MPH treatment, respectively, is mainly due to an increase/reduction of the AMPA currents, confirming the functionality and importance of the AMPARs for the effects observed after treatment with MPH.

## Discussion

In the present study, we compared the effects of single and repeated treatment on the behavioral level in preadolescent rats using the same animals to study the cellular and molecular mechanisms. The results demonstrate that the clinically relevant dose of 1 mg/kg i.p. administered once improves visuo-spatial learning augmenting synaptic plasticity, whereas repeated administration caused the opposite effect, reducing LTP and learning swiftness. These changes were found to be correlated with increased or reduced insertion of GluA1 receptor subunits into the postsynaptic membrane. Further that insertion may not only explain the changes in LTP, but also those concerning short-term plasticity and AMPAR-dependent EPSCs.

### Cognitive Improvement and Increase of Synaptic Plasticity Induced by a Single Dose of MPH

Our present data show that a single dose is sufficient to induce improvement in visuo-spatial learning and an increase in LTP at least up to 48 h after a single MPH administration. However, 6 days after a single dose of 1 mg/Kg we have found no difference to controls. There was a correlation between the behavior and electrophysiological results, since rats that had shown an improvement in visuo-spatial learning also presented a facilitation of approximately 20% of TBS-dependent LTP at the CA3–CA1 synapse. This is comparable to the LTP increase seen after perfusion with 50 μM MPH (Rozas et al., 2015). The increase of LTP by acute MPH in the hippocampus slice has been described earlier (Dommett et al., 2008; Jenson et al., 2015; Rozas et al., 2015) and also with 3,4-methylenedioxymethamphetamine (MDMA; “ecstasy”), an analog of amphetamine (Rozas et al., 2012). Using paired pulse facilitation (PPF) protocols we determined that the MPH-dependent increase in LTP is essentially postsynaptic, since we did not find a difference between controls and rats treated for the interstimulus interval of 50 ms (Figure 2C). In slices of prefrontal cortex from young rats treated with single doses of MPH (1 mg/kg), Urban et al. (2013) also found enhanced induction probability and magnitude of LTP.

The subcellular/molecular changes in the hippocampus observed 48 h after a single MPH dose are the same as those described earlier for plastic changes seen when MPH is present or immediately after MPH application (Rozas et al., 2015). In the present study, we demonstrate that the LTP increase is due to the increase in the rate of externalization of GluA1-containing AMPARs at the cell surface. The acceleration of GluA1 subunits trafficking results from an increase in the phosphorylation at Ser845 of the GluA1 subunit induced by PKA activation, confirming that the phosphorylation of this residue is relevant in the metaplastic effect induced by MPH in the hippocampus. A similar mechanism has also been described in the PFC and nucleus accumbens (Pascoli et al., 2005; Sun et al., 2008, 2005). Other drugs that increase extracellular dopamine, such as MDMA and cocaine, also increase AMPAR GluA1 phosphorylation at Ser845 in the hippocampus, nucleus accumbens, and neostriatum, respectively (Snyder et al., 2000; Rozas et al., 2012; Seo et al., 2013). In addition, Sun et al. (2008), using NAc/PFC co-cultures found that brief incubation with the D1-like agonist SKF 81297 (1 μM, 15 min) increases AMPARs insertion on the extrasynaptic cell surface.

**FIGURE 7:**
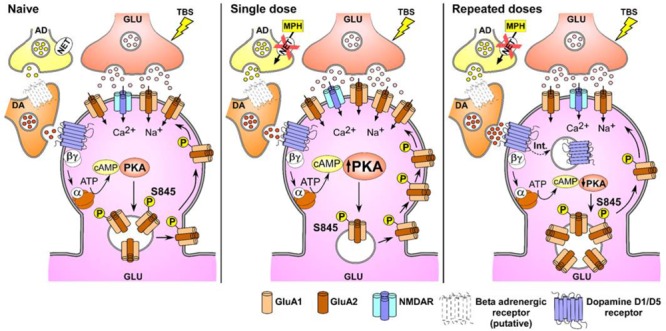
Proposed model to explain the increased and decreased hippocampal synaptic plasticity induced by single vs. repeated administration of MPH. NET, norepinephrine transporter; GLU, glutamatergic neuron; AD, adrenergic neuron; DA, dopaminergic neuron; S845P, Phosphorylated serine residue, dashed arrow: internalization of D1/D5 receptors. Note that the total amount of AMPAR does not change in either condition.

Finally, using whole cell recording from pyramidal cells of the CA1 area of MPH treated rats we found a significant increase in the AMPA-mediated current during short-term plasticity, indicating that the inserted AMPARs were functional. Considering the above evidence, we suggest that the mechanism through which single treatment with MPH modulates synaptic plasticity is similar to the mechanism previously proposed by Rozas et al. (2015) in hippocampal slices superfused with MPH. In that model, MPH increases noradrenaline levels through the blockade of noradrenaline transporters (NET), whose Ki is two times larger than that of DATs (Heal et al., 2009; Koda et al., 2010). Noradrenaline, through β-adrenergic receptor activation, increases extracellular dopamine levels, which, by activation of D1/D5 receptors localized in the postsynaptic membrane, will trigger the trafficking and later insertion of new AMPARs into the extrasynaptic surface of dendritic spines. The mobilization and insertion of new receptors is promoted by phosphorylation of the Ser845 residue of GluA1 subunits of the AMPAR through the activation of the adenyl cyclase-cAMP-PKA cascade. The translocation of these receptors to the synapse during the facilitation of LTP induced by MPH requires stimulation of NMDA receptors by TBS (Wolf and Ferrario, 2010; Rozas et al., 2015), a mechanism that is also at work in other cell types (Oh et al., 2006). In this work, we have complemented our previous model as shown in Figure 7. According to this model, we hypothesize that a single dose of MPH increases the endogenous release of dopamine, which, through D1/D5 receptors activation, increases synaptic plasticity that, in turn, improves spatial learning, as seen in our results.

### Cognitive Impairment and Reduction of Synaptic Plasticity by Repeated Treatment With MPH

As medical treatment of ADHD with MPH consists of daily applications, we assessed the effect of repeated applications MPH on visuo-spatial learning and synaptic plasticity. Contrary to what was found with single doses of MPH, repeated treatment of 1 mg/kg MPH significantly decreased the time to reach the platform in the MWM during the 4th, 5th, and 6th sessions. They also decreased the time rats spent in the quadrant where the platform had been situated during training, suggesting impairment in the retrieval of visuo-spatial memory.

As mentioned above, the effect of MPH on cognitive processes depends on many factors. Interestingly, in our experiments the same dose that, given as single dose favors learning, given repeatedly, impedes it. Repetitive oral administration of 5 mg/kg MPH during 7 days to 21- and 34-day-old rats impaired the capacity of novel object exploration (Heyser et al., 2004), with 5 mg/kg of MPH applied orally equivalent to 1 mg/kg applied i.p. (Gerasimov et al., 2000). Further, Scherer et al. (2010) reported that daily injections of 2 mg/kg MPH for 30 days induces a cognitive deficit affecting the acquisition phase of reference memory and working memory in the MWM, suggesting that prolonged exposure of juvenile rats to MPH significantly reduces learning and spatial memory capacities. Taken together, repeated treatments with MPH can cause cognitive impairment in learning and visuo-spatial memory.

We found that the lesser performance in the orientation task of the MWM was correlated to a smaller LTP, presenting a significant decrease of 20% on the average. The decrease of the TBS-dependent LTP was superseded when superfusing the slices with the D1/D5 receptors agonist SKF38393 without affecting the LTPs of slices prepared from saline-treated rats (Figure 3). Given the absence of any apparent change in the PPF (Figure 3), we believe that the reduction of LTP induced by repeated MPH administration is mainly due to modifications at the postsynaptic level. Although this is the first study showing a reduction of LTP after repetitive doses of MPH, studies obtained with other stimulants such as ecstasy, amphetamine, and methamphetamine are in line with the present results (Ishikawa et al., 2005; Arias-Cavieres et al., 2010; Swant et al., 2010). We have previously shown that young rats treated with 0.2 and 2 mg/kg MDMA twice per day for 6 days not only displayed impaired memory and learning, but also a reduced TBS-dependent LTP in CA1–CA3 synapses (Arias-Cavieres et al., 2010).

We hypothesize that the reduction of hippocampal LTP observed in rats treated repeatedly with MPH is a consequence of a decrease in the translocation of AMPARs in the postsynaptic membrane. Consistent with this view, we found that in slices from rats repetitively injected with MPH showing reduced LTP not only had a specific decrease in the phosphorylation at Ser845 of the GluA1 subunit, but also a lower insertion of new AMPARs into the cell surface. Since lower phosphorylation levels of Ser845 were observed in slices from rats treated with MPH, but without TBS stimulation compared to the control group (Supplementary Figures 4B,C), we propose that repeated MPH administration by itself, in absence of TBS, induces a decrease in the traffic of AMPA receptors into the membrane. Further, there was a decrease of the GluA1 subunit at the cell surface and an increase of the intracellular pool of GluA1 subunit in pieces of hippocampal CA1 derived from rats repeatedly treated with MPH (Figure 5). Both, the decrease of the phosphorylation and insertion of GluA1 subunits in slices with reduced LTPs, was reverted by the D1/D5 receptor agonist SKF38932 (5 μM). However, we also found an increase in the phosphorylation at Ser845 and insertion of GluA1 subunits in slices from saline-treated rats (Figures 4G,H; horizontal lines columns). Since we did not observe an increase of the TBS-dependent LTP by SKF38932, we hypothesize that this agonist increases the traffic of GluA1 subunits to extrasynaptic localizations without mobilizing them to the active sites of the synapse as has been suggested by Sun et al. (2008).

The retention of the GluA1 subunit inside the cell may be explained by a decrease in the rate of translocation into the synaptic membrane or an increase in the rate of internalization of the subunits. AMPARs internalization and increase of the intracellular levels of GluA1 at the NAc in response to repeated treatments with amphetamine have been reported (Brebner et al., 2005; Nelson et al., 2009). Although our results cannot directly discriminate between both processes of trafficking, additional evidence allows us to suggest that the increased intracellular pool of receptors is associated to a decline in the insertion of GluA1 subunits. The superfusion of hippocampal slices with SKF38393, a D1/D5 receptor agonist and activator of the downstream cAMP-PKA cascade, can reverted the MPH-associated increase of the intracellular pool of GluA1 subunits in slices prepared from rats repeatedly treated with MPH. This effect could be associated to an increase in the pool of GluA1 subunits into the cell surface (Figures 5G,H). This is in accordance with the notion that repeated treatment with MPH decreases the activity of adenylyl cyclase-cAMP-PKA dependent cascade (Figure 7). Our results found with the adenylyl cyclase stimulator, forskolin (Figures 5E,F), is compatible with this view. Consistent with the decrease of the number of GluA1 subunits in the membrane, we also found a significant reduction of the AMPA-mediated EPSC and short-term plasticity in slices from rats treated repeatedly with MPH. A decrease in the AMPA-mediated EPSC has also been observed in NAc slices prepared from young mice treated for 5 days with cocaine (Wooters et al., 2011). This reduction is consistent with previous evidence showing that repeated amphetamine administration decreases the levels of GluA1 and GluA2 mRNA in NAc at day 14 of withdrawal time (Lu et al., 1997).

The AMPARs are tetrameric ionotropic receptors constituted of four GluA1-4 subunits, where the GluA2 subunit confers the Ca^2+^ impermeability (Henley and Wilkinson, 2016). It is known that the dynamic regulation of the AMPARs subunit composition is a crucial factor in synaptic functioning (Diering and Huganir, 2018). Although our study was not focused on determining the type of AMPAR involved in the process, based on our evidence and considering that about 81% of AMPARs in the synapses of the hippocampal CA1 are heteromers constituted by GluA1A2 subunits, while an approximately 16% are GluA2A3 (Lu et al., 2009), we propose that the AMPARs that remain on the cell surface after single and repeated dose treatments with MPH are mainly GluA1A2-containing and, to a lower extent GluA2A3-containing AMPARs.

Similar to MPH, forskolin exerted a priming effect on the insertion of GluA1-containing AMPARs (Figures 5E,F). Our results obtained with the application of SKF 38393 also support this view since this D1/D5 agonist superseded the LTP decrease and increased the phosphorylation and insertion of AMPARs into the membrane surface of slices prepared from repeatedly treated rats (Figures 3–5). In line with this, decreases in PKA and adenylyl cyclase activity were found in the striatum of rats treated with repeated doses of MPH (Crawford et al., 1998).

Still another mechanism has been described concerning the action of MPH in cognitive functions. There may be also a role of adrenoreceptors in the changes caused by MPH (Arnsten and Dudley, 2005; Ji et al., 2008). Additional studies should be performed to assess the role of these receptors in the acute and chronic effects of MPH.

Taken together, our findings demonstrate that single and repeated applications of MPH induce opposite effects on both behavior and synaptic plasticity. Single dose administration of MPH induces a cognitive improvement increasing the TBS-dependent LTP by additional mobilization and insertion of GluA1-containing AMPARs into the cell surface. Repeated administrations of MPH on normal preadolescent rats, however, causes learning and memory impairment, decreasing the TBS-dependent LTP by lowering the translocation and insertion of GluA1-AMPARs into the postsynaptic membrane (Figure 7). The mechanisms shown here in preadolescent rats are relevant to better understand the effects of this psychostimulant and possibly its impact in the treatment of ADHD. Furthermore, they may be useful for finding new pharmacological targets that could be used in the treatment of this neuropsychiatric disorder.

## Ethics Statement

All of the protocols dealing with the maintenance and handling of animals were followed as stated in the Bioethical Guidelines of the Universidad de Santiago de Chile and in the National Research Commission guidelines.

## Author Contributions

CC, DC, GU, RD, RP, and CR performed experiments and analyzed data. GU, FP, LC, MZ, and BM designed the experiments. CC, GU, MZ, and BM wrote the paper.

## Conflict of Interest Statement

The authors declare that the researchwas conducted in the absence of any commercial or financial relationships that could be construed as a potential conflict of interest.

## Supplementary Material

FIGURE S1 Methylphenidate (MPH) does not influence swimming speed nor anxiety. There are no significant differences in the average swimming speed between controls (white columns) and treated rats with MPH (black columns) in any session. This was found for both single dose [*F*_(1,60)_ = 0.499, *p* = 0,482; Bonferroni *post hoc* test, *p* > 0.05; *n* = 21; **A**] and repetitive treatments [*F*_(1,60)_ = 0.132, *p* = 0.718; Bonferroni *post hoc* test, *p* > 0.05; *n* = 21; **B**]. In the plus maze no significant difference in the average time of permanence in the open arm between controls (white columns) and treated rats (black columns) measured on the 1th and 6th days, for both, single [days 1 and 6 *F*_(1,36)_ = 0.258, *p* = 0,614, Bonferroni *post hoc* test, *p* > 0.05; *n* = 24; **C**] and repetitive administration [days 1 and 6 *F*_(1,36)_ = 0.506, *p* = 0.50, Bonferroni *post hoc* test, *p* > 0.05; *n* = 24; **D**]. Taken together, these results render it unlikely that the effects of MPH observed were due to locomotor activity or anxiety.

FIGURE S2 Corticosterone levels do not change with repeated doses of MPH. No significant difference was found in corticosterone levels between repeated-treated rats over 6 days (MPH) compared to controls (Control): 47.07 ± 4.08 ng/ml vs. 48.09 ± 3.88 (*n* = 10; *p* > 0.05). To exclude possible effects of MWM training we also measured corticosterone levels in naïve rats without treatment or training. No significant difference was found between these rats [Control (-)] and saline-treated ones (Control): 48.61 ± 5.25 ng/ml vs. 47.07 ± 4.08 (*n* = 10; *p* > 0.05). Data are presented as average ± SEM. Method: Plasma corticosterone levels were determined using a competitive enzyme immunoassay as described in the manual of the Corticosterone ELISA kit provided by Abcam Company; United States. Blood samples were obtained at the end of each experiment and plasma was collect using sodium citrate as an anticoagulant. Samples were centrifuged at 2000 × *g* for 10 min.

FIGURE S3 LTP, phosphorylation and insertion of GluR1 subunits do not changed after of 6 days of a single administration of MPH. **(A)** Time course of the TBS-induced LTP in slices prepared from rats treated with a single dose of saline (open circles) and rats treated with a single dose of MPH (solid circles), 6 days the after administration of MPH. LTP was quantified as in Figure 2. Insets above show representative recordings obtained by averaging 10 traces, at times marked 1 and 2 during the experiments. The small arrow under the traces indicates the presynaptic volley. Values given in parentheses: number of animals and recorded slices, respectively. The arrow indicates TBS. **(B)** Control LTPs (149.3 ± 6.6%; *n* = 6,8; baseline: 100%), LTPs of slices from rats treated with 1 mg/kg MPH (140.1 ± 4.9%; *n* = 4,7). No significant difference was found (*p* > 0.05). **(C,D)** After carrying out the LTP experiments, hippocampal slices were collected and the phosphorylation state analyzed at the Ser845 residue of the GluA1 subunit after 6 days using Western blot technique. **(C)** Immunoblot using an antibody against the phosphorylated Ser845 of GluA1 in Control LTP (+) = slices from saline-treated rats and MPH LTP(+) = slices from MPH-treated rats, and an antibody recognizing the C-terminal end of GluA1 (total GluA1). **(D)** Quantification of Ser845 phosphorylation ratio 60 min after TBS. No significant difference was found between Control LTP (+): 0.56 ± 0.06 and MPH LTP(+): 0.57 ± 0.07 (*n* = 6,6; *p* > 0.05). **(E,F)** In order to determine changes in the number of AMPAR GluA1 subunits in the post-synaptic membrane of the CA1 area of the hippocampus after 6 days, a crosslinking assay was performed. **(E)** Immunoblot using antibody against the phosphorylated Ser845 of GluA1 in Control LTP (+) = slices from saline-treated rats and MPH LTP(+) = slices from MPH-treated rats, and antibody recognizing the C-terminal end of GluA1 (total GluA1). **(F)** Quantification of Ser845 phosphorylation ratio 60 min after TBS. Consistent with our electrophysiological results no significant difference was found in the fraction of cell-surface-associated GluA1 subunits between controls vs. treated rats [Controls LTP(+) = 0.54 ± 0.06 and MPH LTP(+) = 0.51 ± 0.04 (*n* = 6,6; p > 0.05]. These results suggest that a single dose of MPH after 6 days does not mobilize GluA1 subunits from the intracellular pool to the surface.

FIGURE S4 The D1/D5 receptor-agonist SKF38393 does not change TBS-induced LTP in hippocampal slices from animals treated with repeated doses of MPH. **(A)** Time course of TBS-induced LTP in slices prepared from rats treated repeatedly with saline in absence (open circles) or presence (solid triangle) of 5 μM SKF38393; a specific agonist of D1/D5 dopaminergic receptors. The agonist was applied to the bath 10 min before and 10 min after TBS. LTP was quantified as the increase in the slope of the onset of stimulus-evoked fEPSPs (average ± SEM). Insets above show representative recordings obtained by averaging 10 traces, under the conditions at times marked 1 and 2 during the experiments. The small arrow below indicates the presynaptic volley. The values given in parentheses indicate number of animals and recorded slices, respectively. The arrow indicates TBS. **(B)** Quantification of LTPs. No significant difference was found between the LTPs recorded in slices derived from rats treated repeatedly with saline in absence (154.7 ± 0.36%, *n* = 7,9) vs. presence of SKF38393 (157.4 ± 1.98%), *n* = 5,9; *p* = 0.281, Mann–Whitney *U* test). **(C)** Paired-pulse protocols in hippocampal slices prepared from repeatedly treated rats with saline in absence and presence of SKF38393. Inset: synaptic responses evoked by two stimulations applied with interstimulus intervals of 50 ms at times marked 1 and 2 in the experiments in **(A)**. The white columns represent the paired pulse ratio (PPR) of slices from saline-treated rats in absence of SKF pre- (1) and post- (2) application of TBS. The black columns represent the PPR of slices from saline-treated rats in presence of SKF pre- (1) and post- (2) application of TBS. No significant difference in the PPR was observed in any of the conditions analyzed. Control in absence of SKF: 1.19 ± 0.06 (1) vs. 1.13 ± 0.05 (2) (*n* = 7,9). Control in presence of SKF: 1.21 ± 0.01 (1) vs. 1.22 ± 0.01 (2) (*n* = 5,9; *p* > 0.05).
