# Supplementary figures and images for: Single and Repeated Administration of Methylphenidate Modulates Synaptic Plasticity in Opposite Directions via Insertion of AMPA Receptors in Rat Hippocampal Neurons
Source: Front Pharmacol. 2018 Dec 19;9:1485. doi: 10.3389/fphar.2018.01485 (PMC6305740; doi:10.3389/fphar.2018.01485)

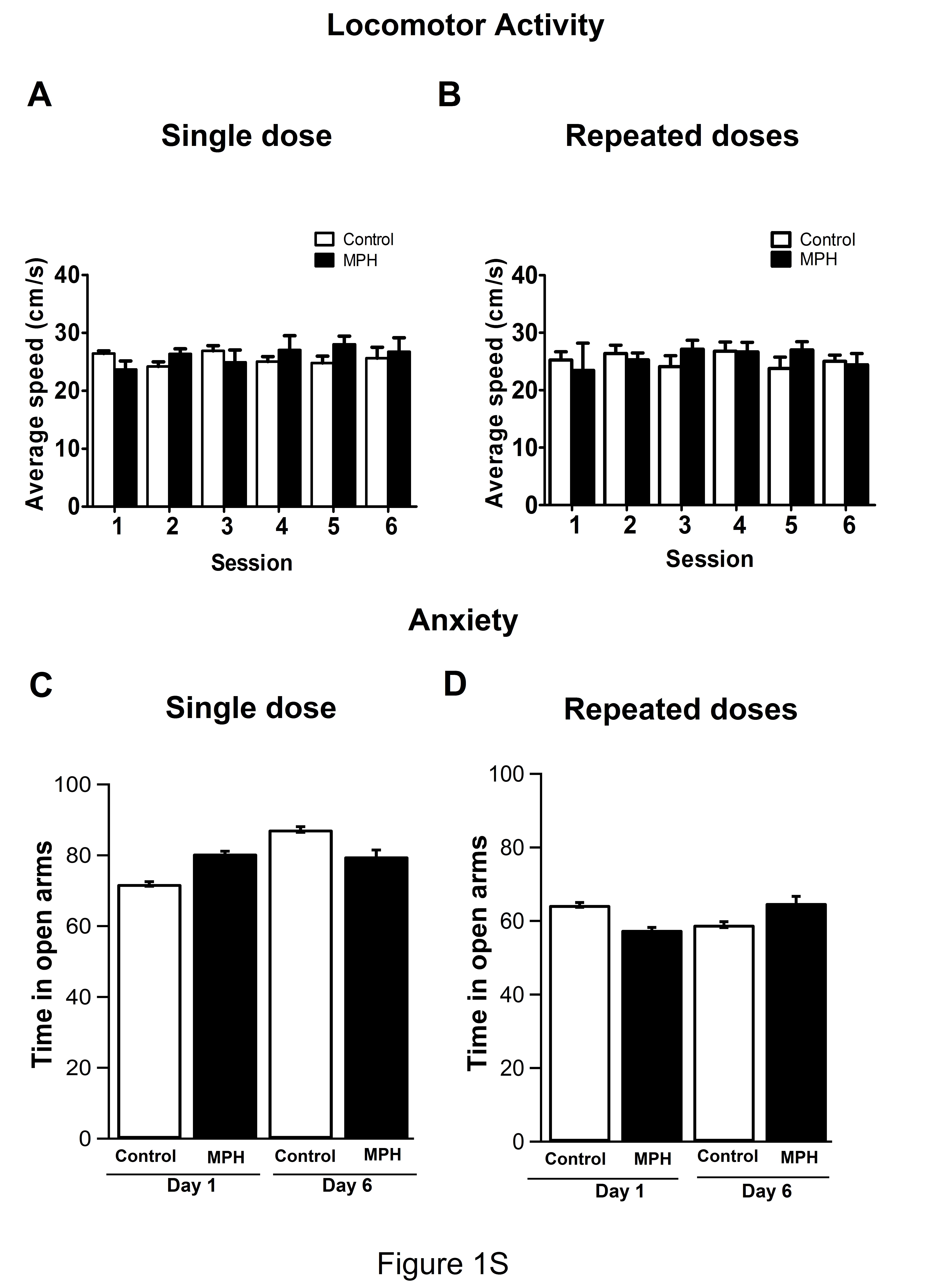

Supplement: FIGURE S1 — Methylphenidate (MPH) does not influence swimming speed nor anxiety. There are no significant differences in the average swimming speed between controls (white columns) and treated rats with MPH (black columns) in any session. This was found for both single dose [F(1,60) = 0.499, p = 0,482; Bonferroni post hoc test, p > 0.05; n = 21; A] and repetitive treatments [F(1,60) = 0.132, p = 0.718; Bonferroni post hoc test, p > 0.05; n = 21; B]. In the plus maze no significant difference in the average time of permanence in the open arm between controls (white columns) and treated rats (black columns) measured on the 1th and 6th days, for both, single [days 1 and 6 F(1,36) = 0.258, p = 0,614, Bonferroni post hoc test, p > 0.05; n = 24; C] and repetitive administration [days 1 and 6 F(1,36) = 0.506, p = 0.50, Bonferroni post hoc test, p > 0.05; n = 24; D]. Taken together, these results render it unlikely that the effects of MPH observed were due to locomotor activity or anxiety. [file Image_1.JPEG]

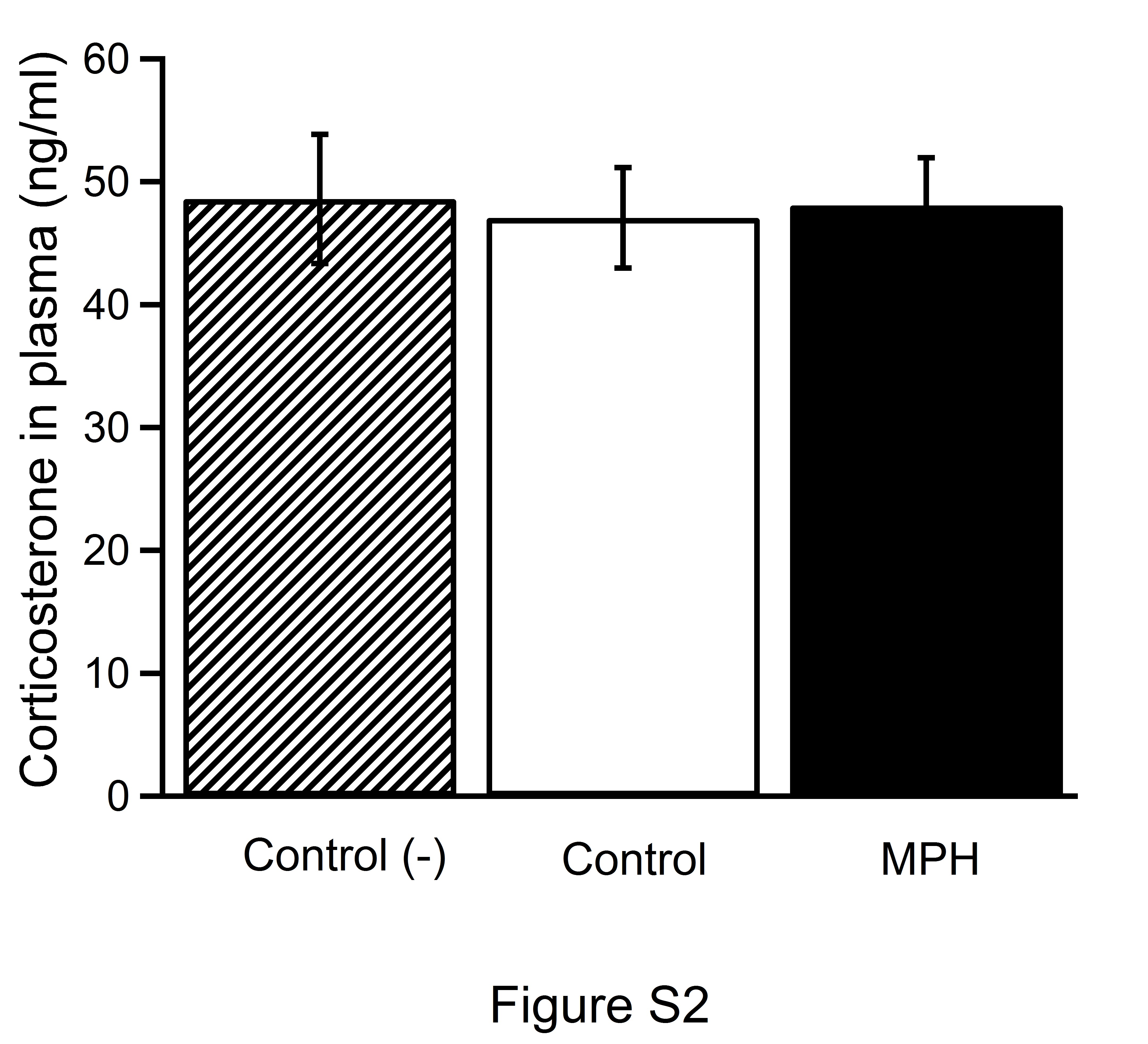

Supplement: FIGURE S2 — Corticosterone levels do not change with repeated doses of MPH. No significant difference was found in corticosterone levels between repeated-treated rats over 6 days (MPH) compared to controls (Control): 47.07 ± 4.08 ng/ml vs. 48.09 ± 3.88 (n = 10; p > 0.05). To exclude possible effects of MWM training we also measured corticosterone levels in naïve rats without treatment or training. No significant difference was found between these rats [Control (-)] and saline-treated ones (Control): 48.61 ± 5.25 ng/ml vs. 47.07 ± 4.08 (n = 10; p > 0.05). Data are presented as average ± SEM. Method: Plasma corticosterone levels were determined using a competitive enzyme immunoassay as described in the manual of the Corticosterone ELISA kit provided by Abcam Company; United States. Blood samples were obtained at the end of each experiment and plasma was collect using sodium citrate as an anticoagulant. Samples were centrifuged at 2000 × g for 10 min. [file Image_2.JPEG]

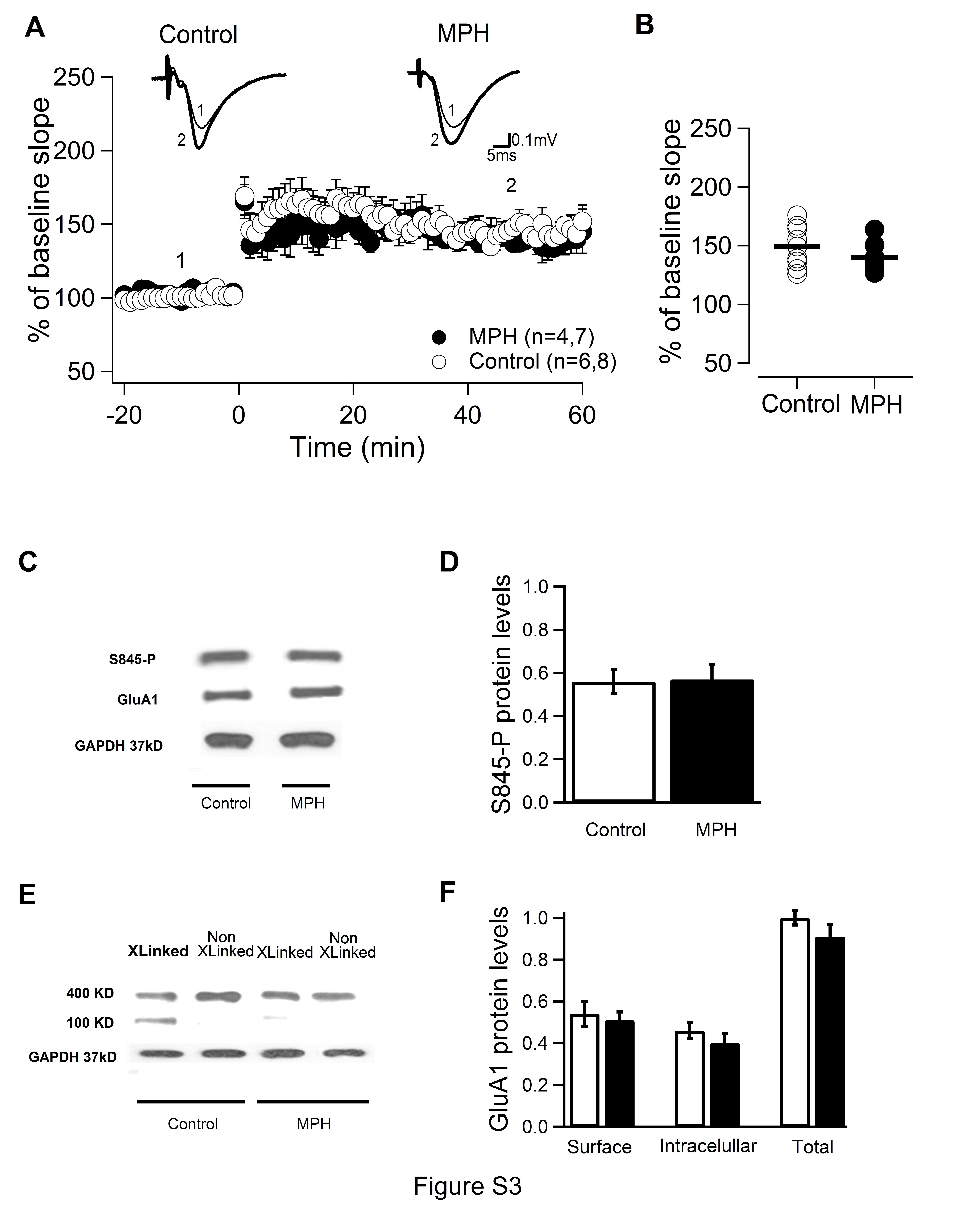

Supplement: FIGURE S3 — LTP, phosphorylation and insertion of GluR1 subunits do not changed after of 6 days of a single administration of MPH. (A) Time course of the TBS-induced LTP in slices prepared from rats treated with a single dose of saline (open circles) and rats treated with a single dose of MPH (solid circles), 6 days the after administration of MPH. LTP was quantified as in Figure 2. Insets above show representative recordings obtained by averaging 10 traces, at times marked 1 and 2 during the experiments. The small arrow under the traces indicates the presynaptic volley. Values given in parentheses: number of animals and recorded slices, respectively. The arrow indicates TBS. (B) Control LTPs (149.3 ± 6.6%; n = 6,8; baseline: 100%), LTPs of slices from rats treated with 1 mg/kg MPH (140.1 ± 4.9%; n = 4,7). No significant difference was found (p > 0.05). (C,D) After carrying out the LTP experiments, hippocampal slices were collected and the phosphorylation state analyzed at the Ser845 residue of the GluA1 subunit after 6 days using Western blot technique. (C) Immunoblot using an antibody against the phosphorylated Ser845 of GluA1 in Control LTP (+) = slices from saline-treated rats and MPH LTP(+) = slices from MPH-treated rats, and an antibody recognizing the C-terminal end of GluA1 (total GluA1). (D) Quantification of Ser845 phosphorylation ratio 60 min after TBS. No significant difference was found between Control LTP (+): 0.56 ± 0.06 and MPH LTP(+): 0.57 ± 0.07 (n = 6,6; p > 0.05). (E,F) In order to determine changes in the number of AMPAR GluA1 subunits in the post-synaptic membrane of the CA1 area of the hippocampus after 6 days, a crosslinking assay was performed. (E) Immunoblot using antibody against the phosphorylated Ser845 of GluA1 in Control LTP (+) = slices from saline-treated rats and MPH LTP(+) = slices from MPH-treated rats, and antibody recognizing the C-terminal end of GluA1 (total GluA1). (F) Quantification of Ser845 phosphorylation ratio 60 min after TBS. Cons [file Image_3.JPEG]

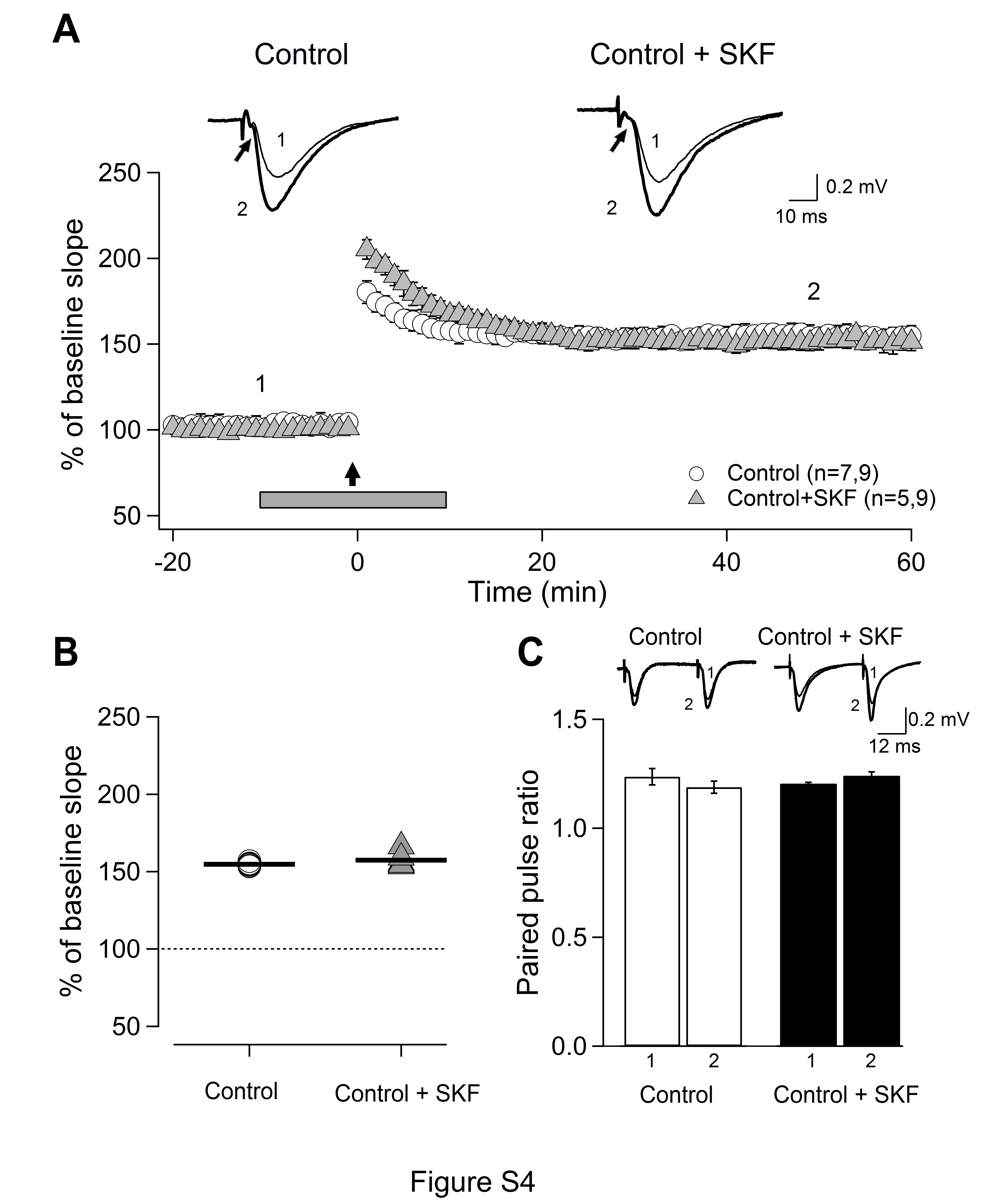

Supplement: FIGURE S4 — The D1/D5 receptor-agonist SKF38393 does not change TBS-induced LTP in hippocampal slices from animals treated with repeated doses of MPH. (A) Time course of TBS-induced LTP in slices prepared from rats treated repeatedly with saline in absence (open circles) or presence (solid triangle) of 5 μM SKF38393; a specific agonist of D1/D5 dopaminergic receptors. The agonist was applied to the bath 10 min before and 10 min after TBS. LTP was quantified as the increase in the slope of the onset of stimulus-evoked fEPSPs (average ± SEM). Insets above show representative recordings obtained by averaging 10 traces, under the conditions at times marked 1 and 2 during the experiments. The small arrow below indicates the presynaptic volley. The values given in parentheses indicate number of animals and recorded slices, respectively. The arrow indicates TBS. (B) Quantification of LTPs. No significant difference was found between the LTPs recorded in slices derived from rats treated repeatedly with saline in absence (154.7 ± 0.36%, n = 7,9) vs. presence of SKF38393 (157.4 ± 1.98%), n = 5,9; p = 0.281, Mann–Whitney U test). (C) Paired-pulse protocols in hippocampal slices prepared from repeatedly treated rats with saline in absence and presence of SKF38393. Inset: synaptic responses evoked by two stimulations applied with interstimulus intervals of 50 ms at times marked 1 and 2 in the experiments in (A). The white columns represent the paired pulse ratio (PPR) of slices from saline-treated rats in absence of SKF pre- (1) and post- (2) application of TBS. The black columns represent the PPR of slices from saline-treated rats in presence of SKF pre- (1) and post- (2) application of TBS. No significant difference in the PPR was observed in any of the conditions analyzed. Control in absence of SKF: 1.19 ± 0.06 (1) vs. 1.13 ± 0.05 (2) (n = 7,9). Control in presence of SKF: 1.21 ± 0.01 (1) vs. 1.22 ± 0.01 (2) (n = 5,9; p > 0.05). [file Image_4.JPEG]
